# Semi-quantitative risk assessment of African swine fever virus introduction in pig farms

**DOI:** 10.3389/fvets.2023.1017001

**Published:** 2023-01-27

**Authors:** Annalisa Scollo, Francesco Valentini, Giorgio Franceschini, Alessia Rusinà, Stefania Calò, Veronica Cappa, Alessandro Bellato, Alessandro Mannelli, Giovanni Loris Alborali, Silvia Bellini

**Affiliations:** ^1^Department of Veterinary Sciences, University of Torino, Torino, Italy; ^2^Istituto Zooprofilattico della Lombardia ed Emilia-Romagna, Sorveglianza Epidemiologica, Brescia, Italy

**Keywords:** African swine fever, semi-quantitative risk assessment, biosecurity, pig, failure mode and effect analysis

## Abstract

A semi-quantitative risk assessment was developed to classify pig farms in terms of the probability of introduction of African swine fever virus (ASFV). Following on-farm data collection *via* a specific checklist, we applied a modified failure mode and effect analysis (FMEA) to calculate the risk priority codes (RPC's), indicating increasing risk levels ranging from 1 to 5. The importance of biosecurity measures was attributed by experts. To consider geographic risk factors, we classified pig farms based on local density of farmed pigs, and on the estimated wild boar population density. The combination of RPC's with geographical risk factors resulted into a final ranking of pig farms in terms of the risk of ASFV introduction. Furthermore, the estimation of frequency and levels of non-compliance with biosecurity measures was used to identify weak points in risk prevention at farm level. The outcome of the risk assessment was affected by choices in assigning non-compliance scores and importance to specific components of biosecurity. The method was applied in 60 commercial farms in major pig production areas in Italy. Furthermore, we applied a reduced version of our checklist in 12 non-commercial/small commercial (≤20 pigs) farms in the northern Apennines. In commercial farms, highest RPC's were obtained for biosecurity measures associated with personnel practices and farm buildings/planimetry. Intervention should be addressed to training of personnel on biosecurity and ASF, to avoid contacts with other pig herds, and to improve practices in the entrance into the farm. Sharing trucks with other farms, and loading/unloading of pigs were other weak points. Fencing was classified as insufficient in 70% of the commercial farms. Among these farms, breeding units were characterised by the lowest risk of ASFV introduction (although differences among median ranks were not statistically significant: *P*-value = 0.07; Kruskal–Wallis test), and increasing herd size was not significantly correlated with a higher risk (Kendall's τ = −0.13; *P*-value = 0.14). Density of farmed pig was greatest in the main pig production area in northern Italy. Conversely, exposure to wild boars was greatest for non-commercial/small commercial farms on the Apennines, which were also characterised by non-compliance with critical biosecurity measures.

## 1. Introduction

African swine fever (ASF) is an infectious haemorrhagic and severe disease in domestic and wild pigs caused by the African swine fever virus (ASFV). The clinical syndromes vary from hyperacute, acute, and subacute to chronic, depending on the virulence of the virus. ASF is a notifiable disease to the World Organization for Animal Health (WOAH) and is one of the major threats to the swine industry worldwide. Its spread into new countries leads to devastating socio-economic losses in the entire swine production sector among others owing to the trade restrictions on animals and animal products (1).

**Figure 1:**
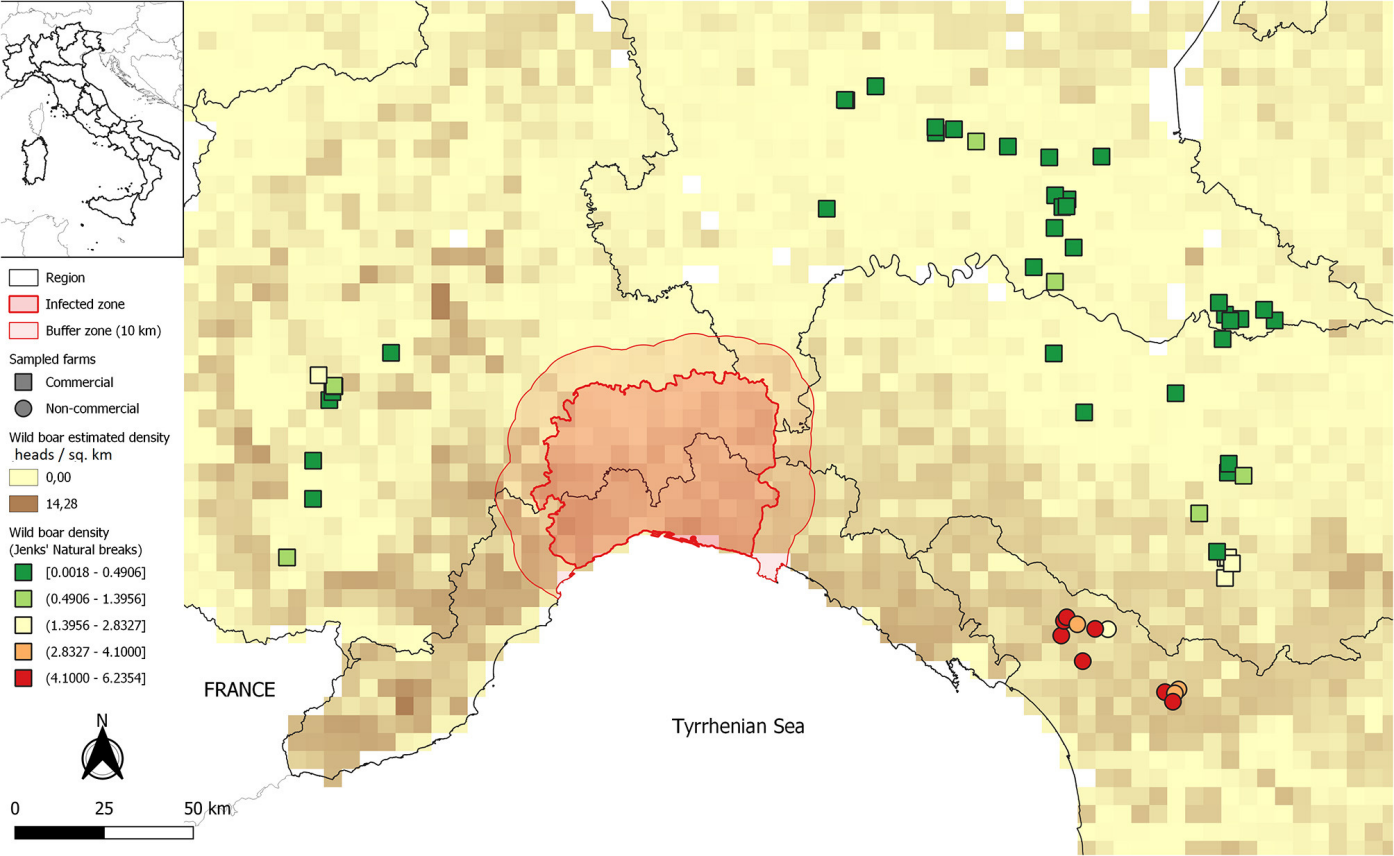
Geographic distribution of visited 72 pig farms, and of the population density of wild boars in Italy. Commercial farms are identified by squares. Non-commercial and small commercial farms in north-western Tuscany are identified by circles. Farm location colour is based on Jenks natural breaks classification method, of all farm types combined, to better visualize variations in wild boar density. The infected area for ASF (4) is reported.

The first report of ASF outside Africa came from in Portugal, in 1957. Epidemics occurred in European and American countries in the following decades. After the eradication of ASF from the Iberian Peninsula in 1995, for several years, the Italian island of Sardinia was the only non-African region where the infection was present (2). The unexpected introduction of ASFV genotype II into the Caucasus in 2007 resulted in an unprecedented geographical spread of the disease. The number of countries or territories reporting the presence of the disease has increased in the last few years, and ASF has officially been notified to the World Organization for Animal Health (WOAH) by member countries from sub-Saharan Africa, Europe, Asia, and the archipelago of the Caribbean region (3). On 7 January 2022, ASFV was confirmed in a wild boar in the province of Alessandria (Piedmont region, northwest Italy), followed by several other cases in the wild boars population up to now, mainly between the Piedmont and Liguria regions^1^ (4) (Figure 1).

The relative importance of different transmission routes and the possible duration of the persistence of ASFV vary across habitats and pig husbandry methods (1, 5, 6). The virus is mainly transmitted by direct contact between infected and susceptible pigs (*via* infectious body fluids and aerosols over short distances between pens) or through the ingestion by susceptible suids of ASFV-contaminated carcasses or pork. The illegal movement of live pigs and pork is considered to be important for the long-distance spread of ASFV (1). Other disease transmission pathways include vehicles and other fomites, such as clothing, footwear, surgical equipment, workers and visitors, slurries, and genetic materials. In certain areas, soft ticks of the genus *Ornithodoros* play a role in transmitting the disease. Wild boars are susceptible to ASF, and in the current European scenario, the disease is endemic in wild boars in several countries and, in the affected areas, they represent a constant threat to domestic pigs.

Since there is no effective vaccine available, the prevention and control of ASF is based on biosecurity and the early detection of the infection by effective surveillance. Recent studies have indicated that insufficient biosecurity measures and ineffective surveillance contribute to virus introduction and spread (7). Biosecurity can be defined as a set of structural, logistical-managerial, and behavioural measures aimed to eliminate or reduce the risk of introduction, establishment, and spread of disease-causing agents in a population (8). Biosecurity measures should be adapted to each disease and farming system. Over the years, checklists were developed to evaluate biosecurity at the farm level. These are based on an objective assessment of measures and may include weighting coefficients, reflecting the relative importance of the assessed parameters. Among these checklists, Biocheck.UGent™ is a risk-based scoring system, which considers the relative importance of all different biosecurity measures to quantify the on-farm internal and external biosecurity (9). Other checklists have been developed, such as the Italian ClassyFarm biosecurity checklist^2^, which extends the collection of information on biosecurity according to Biocheck.UGent™ to include also animal welfare, veterinary antimicrobial use (AMU), antimicrobial stewardship in farms, and inspections at slaughterhouses. Data processing results into a final score for each area of interest, allowing a comparison of the farm results with the average at the national, regional, or local level. Other checklists are the APIQ√®–Australian Pork Industry Quality Assurance Programme^3^, and Japanese BioAsseT (10).

Whereas, the checklists listed above are targeted to general farm biosecurity, disease-specific checklists have been developed for pig farms to evaluate the risk of introduction and spread of *Streptococcus suis* (11) and porcine reproductive and respiratory syndrome virus (PRRSV) (12–14). Two ASF-specific tools are available: the webpage of Vechta University, which allows German pig farmers to perform a self-evaluation^4^, and the outcome-based checklist for ASF-free compartments from WOAH (15). Both checklists, however, include limited information on geographical risk factors. The densities of wild boars, and of farmed pigs at local level were shown as important risk factors for ASFV introduction into farms (16–19). Therefore, the development of a biosecurity checklist, considering ASF-specific transmission routes, as well as risk factors at the geographical level, is necessary for risk assessment and for disease prevention (20, 21).

The evaluation of biosecurity is part of the animal health risk assessment, which is a transparent process for estimating the probability and consequences of the introduction of infectious diseases in free populations. It is based on the reconstruction of phases leading to adverse health outcomes using the best available scientific evidence (22). Important issues related to ASF and biosecurity have been illustrated by several authors. For example, qualitative risk assessment of the introduction of ASFV was applied at the country or continent level when information was limited, and the identification of gaps in knowledge was part of the study's objectives (23). Other authors used a quantitative risk assessment to predict the probability of ASF, and uncertainties in the parameters were included using probability distributions (24). Moreover, a semi-quantitative risk assessment of ASF resulted in the ranking of routes of ASF introduction from wild boars into pig farms by expert elicitation, providing the basis for prioritisation in prevention (25).

The collection of information at the farm level has most often been used to estimate the association between the risk factors and ASF occurrence in the analysis of past epidemics (26–28). Results from these studies provided scientific evidence supporting the adoption of criteria, when assessing the risk of pig farms before ASF occurrence. Such a farm-level risk assessment can be useful for identifying the critical points in biosecurity measures as targets for intervention, and for the classification of establishments for the risk of disease introduction, as provided by the European Animal Health Law as the basis for prevention and control (8).

In this study, we applied a semi-quantitative risk assessment method to classify and rank pig farms in terms of the risk of introduction of ASFV, which takes into account the relative importance of the different transmission pathways. We developed an *ad hoc* checklist for the collection of data on the potential routes of ASFV introduction into pig farms, which were filled during farm visits. To consider the geographical risk factors, we classified the pig farms based on the estimated wild boar population density in the surrounding area. Furthermore, we used an index of local spatial clustering to classify the farms in terms of the domestic pig population density. The data were analysed using a modified failure mode and effect analysis (FMEA) which was previously used to provide a rank of failure modes in the manufacturing industry (29). We adapted such a modified FMEA to identify potential points of failure in the prevention of ASFV introduction in pig farms, taking into account the ordinal properties of biosecurity scores, and their importance. As a result, risk priority codes were obtained for main biosecurity criteria. Their combination with geographical risk factors resulted into a final ranking of pig farms in terms of the risk of ASFV introduction. Furthermore, the estimation of frequency and levels of non-compliance with biosecurity measures was used to identify weak points in risk prevention at farm level. An example of application of the checklist to pig farms in northern Italy is presented.

## 2. Materials and methods

### 2.1. Development of a biosecurity scoring system

The ASF-specific questionnaire developed in the present study aims to describe the complete biosecurity situation in a pig herd. Its development was based on the main biosecurity principles listed in Dewulf and Immerseel (7), Biocheck.UGent™ (9), and the ClassyFarm biosecurity checklist for the Italian Veterinary Authority.^5^ Other biosecurity principles more specific for ASF were introduced from the prescriptions listed in the European Commission working document SANTE/7113/2015—Rev 12^6^ and Commission Implementing Regulation (EU) 2021/605 (30). All the ASFV transmission routes were considered, such as direct contact transmission, movements of animals, semen, ova, embryos, food-borne transmission (e.g., water hygiene, swill feeding), indirect transmission (e.g., personnel, wild birds, insects, environmental enrichments, equipment, rodents, or pets), and environment (e.g., cleaning and disinfecting the barn) (1, 26–28). The final checklist consisted of 98 questions (items) with dichotomous answers. The objective of a checklist with dichotomous answers was the collection of factual observations, excluding subjective opinions. The 98 items included in the checklist were grouped into 24 sub-criteria and, subsequently, into six main biosecurity criteria. The number of sub-criteria contributing to each main criterion varied from three to five (Table 1).

**Table 1:** Main criteria and sub-criteria of the ASF specific checklist and the importance score of each sub-criterion assigned by the experts' opinion.

	**Main criteria**	**Sub-criteria**	**Importance score (95% CL's)**
A	Personnel	A1 Entrance of personnel into the farm	4 (3, 5)
		A2 Contact of personnel with other pigs and wild boar hunting	5 (5, 5)
		A3 Food introduction by personnel	2 (2, 3)
		A4 Personnel training	3 (2, 5)
B	Animal introduction and management	B1 Health/feeding/breeding status of introduced pigs	5 (5, 5)
		B2 Number of farms of origin of the introduced pigs	4 (3, 5)
		B3 Management of animals with an impaired growth	3 (3, 4)
C	Animal shelters management	C1 Quarantine	3 (2, 5)
		C2 Internal animal flow and cleaning procedures	4 (3, 5)
		C3 Vaccine prophylaxis and treatments for other infectious diseases	1 (1, 1)
		C4 Structure and buildings	5 (4, 5)
		C5 Dead pigs' management	2 (2, 3)
D	Animal transport vehicles	D1 Live animal transport vehicles	5 (3, 5)
		D2 Live animal unloading/loading	3 (2, 5)
		D3 Carcass disposal	5 (3, 5)
		D4 Equipment and tools for loading/unloading live animals	4 (2, 5)
E	Material management: feed, slurry, and other vehicles	E1 Procedures for loading/unloading of feed and materials	4 (3, 5)
		E2 Feed and materials storage	3 (2, 5)
		E3 Slurry management	2 (2, 5)
		E4 Vehicles for loading/unloading feed and materials	5 (4, 5)
F	Buildings and farm planimetry	F1 Farm perimeter barriers	5 (4, 5)
		F2 Other animals and disinfection procedures	5 (4, 5)
		F3 Pest and rodent control	3 (2, 3)
		F4 Visitors	4 (3, 4)

The importance ranges from 1 (very low) to 5 (very high). 95% confidence limits (CL's) were obtained by bootstrapping.

### 2.2. Farm categorization method

#### 2.2.1. The importance score: Assignment of importance to different biosecurity sub-criteria

Given that not every ASFV transmission pathway has the same efficiency, biosecurity measures are not equally important in protecting the health of farm animals. For example, it is well known that direct contact between animals (e.g., purchase of live animals, possibility of free range of pigs) poses a higher risk, whereas indirect contact (e.g., transmission of pathogens by fomites, contact with infected material) is less efficient in the transmission of pathogens (31). To establish a hierarchy of importance of the 24 sub-criteria within the six main criteria, the Borda method was used (32). Eight experts from countries affected by ASF, with experience in pig management and ASF control, assigned an importance score ranging from 1 (least important) to 5 (most important) to each of the sub-criteria within each of the six main criteria with respect to its relevance in reducing the risk of ASF introduction into the farm. A modified Borda method was used to obtain a summary importance score for each sub-criterion as the sum of the scores assigned by each expert:


$$\begin{array}{l} I_{b}(x)=\sum_{i=1}^{m}I_{i}(x) \end{array}$$

Where:

*I*_*i*_(*x*) is the importance score assigned to sub-criterion x by the *i*-th expert, and *m* is the number of experts (in this case, *m* = 8). The most important sub-criterion x^*^ is that with the highest Borda score, as shown below:


$$\begin{array}{l} I_{b}\left(x^{*}\right)=\max_{x\in s}\left\{I_{b}(x)\right\} \end{array}$$

where *S* is the set of compared sub-criteria, which are part of each of the main criteria. The most important sub-criterion was assigned a score of 5; the scores of the other sub-criteria were subsequently calculated in decreasing order, until a score of 1, which was assigned to the least important sub-criterion. In our application, the Borda method was modified to allow for ties in the importance scores, which were assigned by experts to each sub-criterion. To report variability in the attribution of importance scores to sub-criteria by the eight experts, and its consequences on the summary importance score *I*_*b*_, as estimated by the modified Borda method, we obtained 95% confidence limits by a bootstrap approach. In particular, for each sub-criterion, we randomly sampled, for 10^4^ times, the eight importance score assigned by the experts (*sample* function in the R software, specifying “size = 8”, and “replace = TRUE”) and calculated *I*_*b*_. By sampling with replacement, the importance score assigned by a particular expert could be selected more than once to be part of each random sample of scores. As a consequence, a greater variability of scores resulted into more variables *I*_*b*_ estimates. The 2.5th, and 97.5th percentiles of the distribution of those 10^4^ *I*_*b*_ estimates were used as the lower, and upper limits of the 95% confidence interval.

During the evaluation of the sub-criteria by the expert panel, some of the 98 items were considered of crucial importance for biosecurity against the introduction of ASFV in pig farms, and those were defined as *critical items* (Table 2).

**Table 2:** List of the 9 “Critical items” selected by the expert's panel, with reference to the sub-criterion in which they are included.

**Critical item**	**Critical item (sub-criterion)**	**N (and %) of non-compliant commercial farms (*n* = 60)**	**N (and %) of non-compliant non-commercial and small commercial farms (*n* = 12)**
1	Change of clothes and footwear is carried out (A1)	2 (3.3)	11 (91.7)
2	The staff has no other pigs (A2)	14 (23.3)	10 (83.3)
3	Staff has no contact with other pig farms (A2)	38 (63.3)	12 (100.0)
4	Staff does not engage in wild boar hunting activities (A2)	6 (10.0)	10 (83.3)
5	Animals are not fed catering waste, canteen waste, or household leftovers (swill feeding) (B1)	0 (0.0)	2 (16.7)
6	While loading animals, transporters help inside the truck, but never enter any clean farm area, which is clearly demarcated (D4)	2 (3.3)	
7	Clothing provided to transporters is company or freshly laundered, and boots are company issued (D4)	7 (11.7)	
8	There is an external fence for the entire farm perimeter that prevents the entrance of wild animals and visitors (F1)	42 (70.0)	12 (100.0)
9	Disinfectants with proven efficacy against ASF are available (F2)	0 (0.0)	12 (100.0)

#### 2.2.2. The non-compliance score

Each of the 24 sub-criteria was assigned a non-compliance score, ranging from 1 (high compliance) to 5 (low compliance), based on the application of the checklist during the on-farm visits. Several items contributed to the score of each sub-criterion, and each of them allowed two possible answers: “yes”, indicating compliance with biosecurity; “no”, indicating non-compliance. The increasing non-compliance score of each sub-criterion was calculated based on the decreasing proportion of “yes” answers to the items in that sub-criterion, as shown in Table 3. In few cases, a sub-criterion included items allowing five mutually exclusive answers, corresponding to an increasing order of non-compliance levels (e.g., sub-criteria B2, and B3, see Supplementary material). In these cases, it was possible to respond only one of these answers, and the corresponding level, from 1 to 5, was assigned as the non-compliance score for that sub-criterion.

**Table 3:** Description of the sub-criterion non-compliance scoring system.

**Sub-criterion non-compliance score**	**Description**
1	All items are satisfied
2	Between 62.6 and 99.9% of the items are satisfied
3	Between 37.6 and 62.5% of the items are satisfied
4	Between 0.1 and 37.5% of the items are satisfied
5	No items are satisfied, or at least one “critical item” is not satisfied

If one of the critical items was not satisfied, the corresponding sub-criterion was assigned the maximum non-compliance score of 5, regardless of the answer to the other items belonging to the same sub criterion.

#### 2.2.3. Calculation of the risk priority codes by failure modes and effect analysis

The importance and non-compliance scores of the sub-criteria were used to calculate a risk priority code (RPC) for each of the six main criteria for each pig farm, using modified failure modes and effect analysis (FMEA), as shown below:


$$\begin{array}{l} RPC\left(a_{i}\right)=\;Max_{j\;}\left\{Min\;\left[\left(Ig_{j}\right),\;g_{j}\left(a_{i}\right)\right]\right\} \end{array}$$

Where:

*RPC*(*a*_*i*_) is the Risk Priority Code for the criterion *a*_*i*_ (with *i* = 1,…, 6);

*g*_*j*_ (*a*_*i*_) is the non-compliance score for each sub-criterion *j* (with *j* = 1,…, n) included in the criterion *a*_*i*_ (calculated as in Table 3);

*I*(*g*_*j*_) is the importance score of each sub-criterion *g*_*j*_, included in criterion *a*_*i*_, as estimated using the Borda method;

*Max*_*j*_is the maximum of the minimum (*Min*) between the non-compliance score for sub-criterion *g*_*j*_ (resulting from the checklist's results) and the importance score that was assigned to sub-criterion *g*_*j*_.

**Figure 2:**
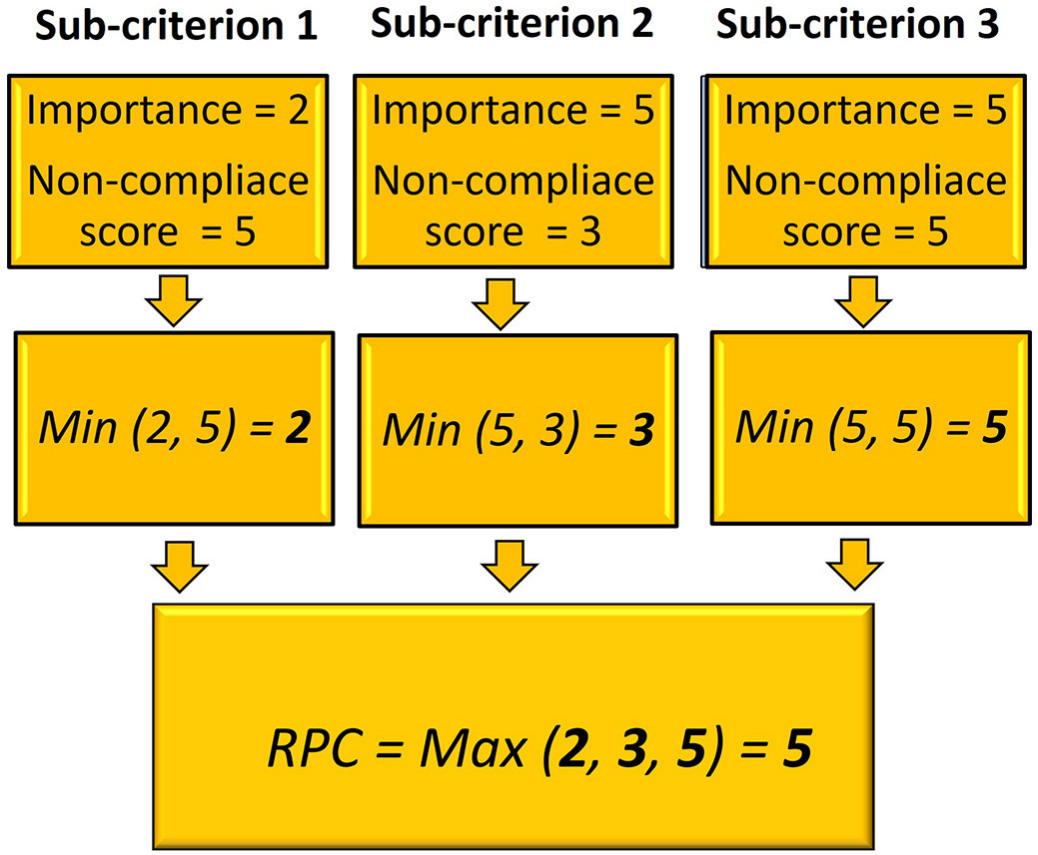
Example of calculating the Risk Priority Code (RPC) for one of the main criteria, in a hypothetical pig farm, using the modified failure mode effect analysis. Sub-criterion 1 was assigned an importance score = 2 by the 8 experts. While, in the assessment of biosecurity on the farm by completing the checklist, the veterinarians assigned a high non-compliance score to this sub-criterion (score = 5), indicating low biosecurity. In the calculation, sub-criterion 1 contributed to the overall RPC with a value of 2, which is the minimum between 5 and 2. Sub-criterion 2, on the other hand, was assigned a high importance (5) and a moderate non-compliance score (3). Therefore, according to the procedure just described sub-criterion 2 was assigned a score of 3. Finally, sub-criterion 3 was assigned a high importance = 5 and a high non-compliance score (5). Therefore, sub-criterion 3 contributed 5 to the final result. In fact, the overall RPC for the main criterion, including sub-criteria 1 to 3, was equal to 5, which was obtained as the maximum of the three minimums: 2, 3 and 5.

This equation corresponds to the second analysis model described by Franceschini and Galetto (29). The aim is to assign a high RPC for a given criterion (*a*_*i*_) to those farms that had the highest non-compliance score (corresponding to low biosecurity) on the most important sub-criteria. As an example, a sub-criterion which has been assigned a non-compliance score of 4 and a low importance score (i.e., 2) would be considered of value 2, as the minimum between 4 and 2. Therefore, the contribution of this sub-criterion to the RPC will be limited. In contrast, if the importance of the sub-criterion was 5, a value of 4 would have been chosen (being the minimum between 5 and 4), and this sub-criterion would have contributed more to the RPC. Indeed, the final RPC of each of the six main criteria was calculated as the maximum value among the scores of all sub-criteria. A graphical description of the calculation of RPC is shown in Figure 2.

#### 2.2.4. Geographical risk factors

Given the major role of wild boars in maintenance and transmission of ASFV in Europe (16), the pig farms were classified based on the estimated wild boar population density at the farm locations. A high-resolution raster map of predicted wild boar densities across most of Eurasia was obtained (33), and imported into the R software (*raster* function, *raster* package). Predicted wild boar densities, corresponding to the locations of examined pig farms, was obtained by the *extract* function. Subsequently, farms were classified by Jenks natural breaks of the estimate, to obtain five ordinal levels of increasing risk of exposure to wild boars, for consistency with the five levels of RPC.

Previous research showed that population density of farmed pig was associated with the occurrence of ASF (17–19). The risk of transmission of ASFV between domestic pigs is a function of the distance between farms and can be modelled by transmission kernels (34). To classify farms also in terms of pig population density, we calculated a modified G statistic (35), as an index of local spatial density as shown in the equation below:


$$\begin{array}{l} G_{i}=\frac{\underset{j}{\sum }w_{ij}x_{j}}{\underset{j}{\sum }x_{j}} \end{array}$$

where *G*_*i*_ is an index of local density of pigs around the visited farm *i*; *x*_*j*_ is the number of pigs in each of the other pig farms *j*; and *w*_*ij*_ is a distance kernel (equation below):


$$\begin{array}{l} w_{ij}=\;\frac{k_{0}}{1+{\left(\frac{h_{ij}}{r_{0}}\right)}^{\alpha }} \end{array}$$

*h*_*ij*_ is the distance between the sampled farm *i* and each of the other near farms _j_; *k*_0_ is the value of *w*_*ij*_ when *h* = 0; *r*_0_ is the distance at which *w*_*ij*_ = 0.5 *k*_0_; and α is the kernel shape parameter. To obtain a smooth decay of local density with increasing distance from other pig farms, we assigned the values of *k*_0_ = 1, *r*_0_ = 0.55 m, and α = 2.27. Such kernel parameters were previously estimated by Boender et al. (36) during the classical swine fever epidemic in the Netherlands, in 1998, and subsequently proposed for ASF by EFSA (34). Farms were subsequently classified by Jenks natural breaks of *G*_*i*_, to obtain five ordinal levels of increasing risk of local density of domestic pigs. All the pig farms were included in the kernel calculation, although, due to the specific kernel shape, only farms within a certain distance influenced the density weight. Spatial analysis was performed by the R software, version 4.1.2, whereas geographic representation, and Jenks natural breaks of the estimates were obtained using QGIS 3.16.2 Hannover Edition.

#### 2.2.5. Overall risk ranking of pig farms

Each examined pig farm was attributed ordered scores (from 1 to 5) for a total of eight indicators: six RPC's for criteria, which were estimated from the on-farm checklist, and two geographical risk indicators, corresponding to wild boar population density, and local density of domestic pigs. To obtain an aggregated risk index, we calculated, for each farm, the counts of decreasing values of those eight risk indicators, from counts of 5 to counts of 1. Subsequently, a risk rank was assigned to each farm, by sorting them in a decreasing order. In this way, highest risk was attributed to those farms which were characterised by the greatest frequency of RPC's = 5. Then, among farms with the same number of 5 s, the one with the greatest number of 4 s was classified at the highest risk. Then, the counts of 3 s, and of 2 s were considered. An overall ranking of farms, ordered from the farm at the highest risk of ASFV introduction (rank = 1) to the lowest risk was obtained.

Limited to commercial farms in major pig production areas in Italy, non-parametric correlation of risk rank and herd size was estimated by Kendall's τ, using the *KendallTauB* function of the *DescTools* package in the R software. Kendall's τ value is appropriate for estimating the correlation between ordinal variables in the presence of ties. Differences among median risk ranks for different production phases were tested by Kruskal Wallis test (*kruskal.wallis* function, R software). See below for definition of herd size and production phases.

### 2.3. Data collection

The farm data collection was carried out mainly in Lombardy, Emilia-Romagna, and Piedmont, the three regions where 77.2% of Italian commercial pig farms are located.^7^ Moreover, non-commercial and small commercial pig farms (pig farms with a maximum of 20 animals) were also visited in Tuscany, in a Northern Apennine area, ~150 km from the Italian ASF-infected area (4). In this second sample of farms, we applied a reduced version of our checklist, not including items which are typical of commercial pig production (Table 2, Supplementary Table S1). The inclusion of non-commercial/small commercial farms in the study must, therefore, be considered as preliminary to more in-depth investigations on these types of pig farms.

We selected pig farms based on the farmers' availability from a list provided by seven pig veterinary practitioners and two official veterinarians. Prior to the assessment, we provided all farmers with a comprehensive explanation of the aims and procedures of the study and obtained informed consent.

Three trained veterinarians who participated in the creation of the checklist carried out the farm visits from March through December 2021. To improve the harmonisation of data collection, the three assessors previously discussed all 98 items and agreed upon written guidelines for filling the checklist. Moreover, if any doubt emerged, the three assessors collectively discussed and took decisions on answers to any specific item.

For each farm, the following general information was collected prior to the biosecurity assessment: geographical coordinates, type of farm (commercial, non-commercial/small commercial), production phase (breeding; post-weaning—from weaning to ~30 kg of body weight; fattening—from ~30 kg of body weight to slaughter; not specialized—more than one production phase on the same farm), production cycle (closed, open, and semi-closed), and herd size (in case of post-weaning and fattening sites: number of farmed pigs present the day of the visit; in case of breeding farms: number of productive sows present the day of the visit). The data were collected through direct observation and face-to-face interviews with the farmers. As suggested by Dewulf and Immerseel (7), it was decided to first visit the farm in order to make a visual assessment of the situation, and then fill the questionnaire with the farmer to simplify and speed up the assessment. Depending on the farm type, it generally took 30 min to 1 h to complete the checklist. During the on-farm biosecurity assessments, the assessors always acted according to good biosecurity practices.

## 3. Results

The checklist was filled in for 60 commercial pig farms and 12 non-commercial and small commercial (≤20 heads) farms. Among the commercial farms, 53 (88.3%) were in Lombardy, Emilia-Romagna, and Piedmont. A limited number of commercial farms were also visited in Umbria (*n* = 3), Abruzzo (*n* = 2), Apulia (*n* = 1), and Veneto (*n* = 1). These additional farms belonged to companies involved in Lombardy and Emilia Romagna. The 12 non-commercial and small commercial pig farms involved in this study were located in northern Tuscany. Thirty-three (55.0%) of commercial farms were fattening farms, six (10.0%) were post-weaning sites, whereas 11 (18.3%) were breeding sites. The other 10 farms (16.7%) were not specialized in a specific productive phase, and included both the post-weaning and fattening phases. The median of heads reared in the commercial farms was 1,915 (minimum = 50 heads; Q1 = 1122; Q3 = 3631; maximum = 42,000).

### 3.1. The importance scores

The importance scores assigned by the experts' panel to each sub-criterion, together with 95% C.L.s, are reported in Table 1. The list of the items that the experts considered as critical for specific biosecurity in the case of ASF consisted of nine of the 98 items, as shown in Table 2.

### 3.2. The non-compliance scores

Details of the non-compliance scores of each of the 98 items are reported in the Supplementary Table S1.

#### 3.2.1. Main criterion A: Personnel

**Figure 3:**
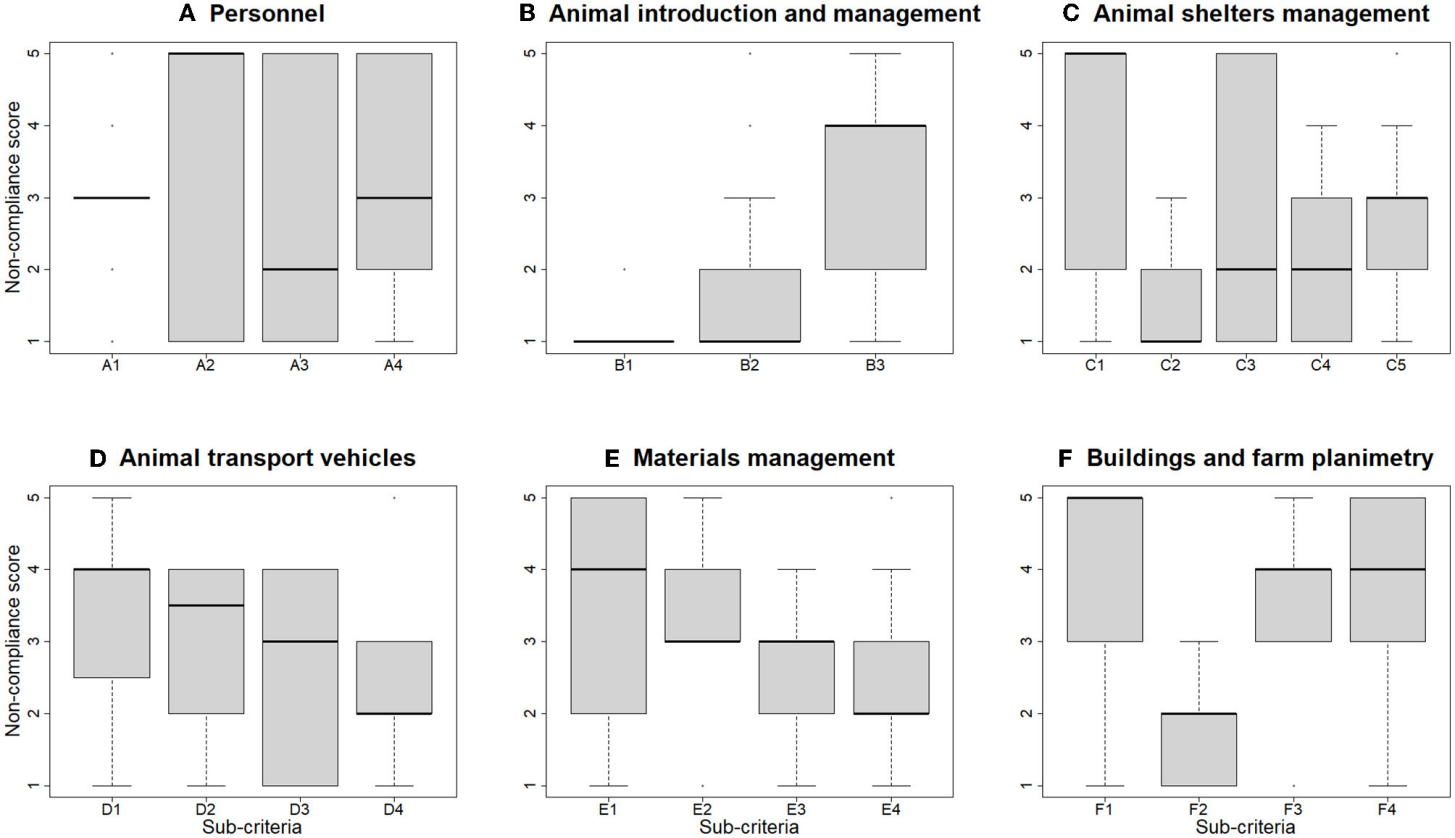
Boxplot of the distribution of non-compliance score for each sub-criterion, obtained by evaluation of 60 commercial pig farms in Italy. In the boxes, the thick horizontal line represents the median non-compliance score; the base and the top of the boxes are the first and third quartiles, respectively. Whiskers extend to a maximum of 1.5 times the interquartile range. Points represent extreme values.

Four critical items were identified by the expert panel among the main criterion A (Table 2). In commercial farms, negative answers to critical items 2 (the staff had no other pigs, in 23.3% of farms) and 3 (the staff had no contact with other pig farms, 63.3%) resulted into a relatively frequent non-compliance score = 5 for sub-criterion A2 (Figure 3). Non-compliance to sub-criterion A3, was associated to 36.7% of the farms where the answer was “staff may introduce food in the farm, and there is no indication to the area in which it must or may be consumed”. Regarding sub-criterion A4, in 28.3% of the farms a non-compliance score of 5 resulted from the selection of the option “the farmer and staff are either not trained at all on biosecurity and the risks of introducing ASF, or there is no clear evidence of courses”. In 30.0% of the farms, non-compliance score was 3 for the item “only a portion of the staff working on the farm is trained in biosecurity and the risks of introducing ASF during the last year”. Overall, 71.7% of the farms had some workers who did not receive any training on biosecurity and the risk of introducing ASF. A high frequency of non-compliance score of 3 for sub-criterion A1 was the result of 70% of the farms having an improper access area, with overlapping clean and dirty areas. Moreover, personnel do not take a shower before entering in 90% the farm, and 93.3% do not have a Danish entry (i.e., a bench or other physical barrier that totally separates the dirty and clean areas and remember the personnel the threshold).

Considering non-commercial and small commercial farms, most of the farms were non-compliant to all four critical items belonging to main criterion A (Table 2). The non-compliance score for sub-criteria A1, A3, and A4 was 5 for the majority of the farms (>75.0%).

#### 3.2.2. Main criterion B: Animal introduction and management

Only one critical item was identified by the expert panel for the main criterion B (animals were not fed by swill feeding), and all commercial farms were compliant, whereas two non-commercial/small commercial farms were non-compliant (16.7%, Table 2). In general, biosecurity associated with the introduction of animals and related management showed a good level of compliance for both commercial and non-commercial/small commercial farms. Considering commercial farms (Figure 3), the most frequent non-compliance score for the sub-criterion B1 and B2 was 1 (i.e., the lowest risk of introduction of ASF); within sub-criterion B1, the totality of the commercial farms knew the health status for ASF of all animals prior to their introduction into the herd, correctly identified all the animals on the farm, accurately registered all animal movements both in/out and within the holding structures, and banned swill feeding. Regarding the sub-criterion B2, the majority of the commercial farms (65.0%) always introduced animals from the same farm of origin during the year. Considering non-commercial and small commercial farms, the frequency of non-compliance score 1 was greater for all three sub-criteria.

#### 3.2.3. Main criterion C: Management of animal shelters

No critical items were identified by the expert panel in main criterion C. The assessment of biosecurity practices associated with quarantine of newly introduced animals (sub-criterion C1) was applicable only to 20 commercial farms, as the remaining 40 farms applied all-in/all-out practices. A non-compliance score of 5 was assigned to 55.0% of commercial farms (Figure 3). Cleaning and disinfection practices were carried out satisfactorily (sub-criterion C2). The importance of proper carcass management and disposal appeared to be, in general, understood by farmers, and a non-compliance score of 5 was recorded in only six farms (10.0%, C5). All the non-commercial/small commercial farms were assigned a non-compliance score of 4 to sub-criterion C3 and a non-compliance score of 2 to sub-criterion C4, whereas sub-criteria C1, C2, C5 were not included in the checklist for these types of farms.

#### 3.2.4. Main criterion D: Animal transport vehicles

Two critical items were identified by the expert panel in main criterion D, and a few commercial farms were non-compliant (Table 2). Biosecurity practices during the transport of live animals through vehicles (sub-criterion D1) and their loading and unloading (sub-criterion D2) were not optimal, resulting in a non-compliance score of 4 for both in at least 30 (50.0 %) commercial farms (Figure 3, see Supplementary material for details). In 84.7% of the farms, vehicles were shared with other pig farms, in 75.0% a loading/unloading bay was not present, and in 69.5% no special gates were in place to prevent animals from returning to the barn. Notably, in non-commercial/small commercial farms, the majority of the farms showed a non-compliance score of 5 regarding sub-criterion D1, and of 4 to sub-criterion D2. Carcass disposal (sub-criterion D3) showed heterogeneous results in commercial farms; in 50.0% of these, the truck for the removal of carcasses entered the farm area (Figure 3). For sub criterion D4 (equipment and tools for loading/unloading live animals) compliance was generally greater than for other sub criteria of criterion D, even though disinfection of animal loading bay was not carried out after every usage in 71.1% of farms. Sub-criteria D3, D4 were not included in the checklist for non-commercial/small commercial farms.

#### 3.2.5. Main criterion E: Material management (feed, slurry, and other vehicles)

No critical items were identified by the expert panel in main criterion E. The practices in loading and unloading feed and other materials (sub criterion E1) were unsatisfactory in 40.0% of commercial farms (median score was 4, Figure 3). In particular, the most frequent negative answer was recorded for the item associated to the common treading of the material loading/unloading bays by internal personnel and external operators without dedicated clothing and footwear (90.0% of the commercial farms, Supplementary Table S1). Feed and material storage (sub criterion E2) resulted in non-compliance score ≥3; 81.4% of the farms were non-compliant with the storage of feed, forage, bedding, or environmental materials for at least 30 days before use. Regarding sub-criterion E3, the entrance for slurry transport operations was not separated from the access of the pigs' area in 65.5% of commercial farms (Supplementary Table S1), and the median non-compliance score was equal to 3 (Figure 3). The management of vehicles transporting materials (sub criterion E4) was satisfactory in the majority of commercial farms, with a median non-compliance score of 2; however, in 54.5% of farms, the vehicles were not disinfected on a dedicated area before access to the farm. In contrast, 100.0% of non-commercial/small commercial farms had a non-compliance score of 5 for sub-criterion E4, whereas sub-criteria E1, E2, E3 were not included in the checklist for these types of farms.

#### 3.2.6. Main criterion F: Buildings and farm planimetry

The panel of experts identified two critical items belonging to the main criterion F (Table 2). In 70.0% of commercial farms and 100.0% of non-commercial/small commercial farms the fencing was incomplete (critical item n. 8, Figure 3, sub-criterion F1). Furthermore, the farming area of 71.7% of commercial farms was not surrounded by an asphalted zone (Supplementary Table S1). Conversely, critical item 9 (“disinfectants with proven efficacy against ASF are available”, part of sub criterion F2) was always satisfied. Pets were present in 60.0% of the commercial farms. Pest management in commercial farms was often suboptimal (sub-criterion F3), rodent control was usually self-managed (85.0% of farms), and no farm had insect and bird control plans in place (median non-compliance score = 4, Figure 3). In non-commercial and small commercial farms, sub-criteria F2, F3, and F4 showed a non-compliance score of 4 or greater in all farms.

### 3.3. The RPC's

**Figure 4:**
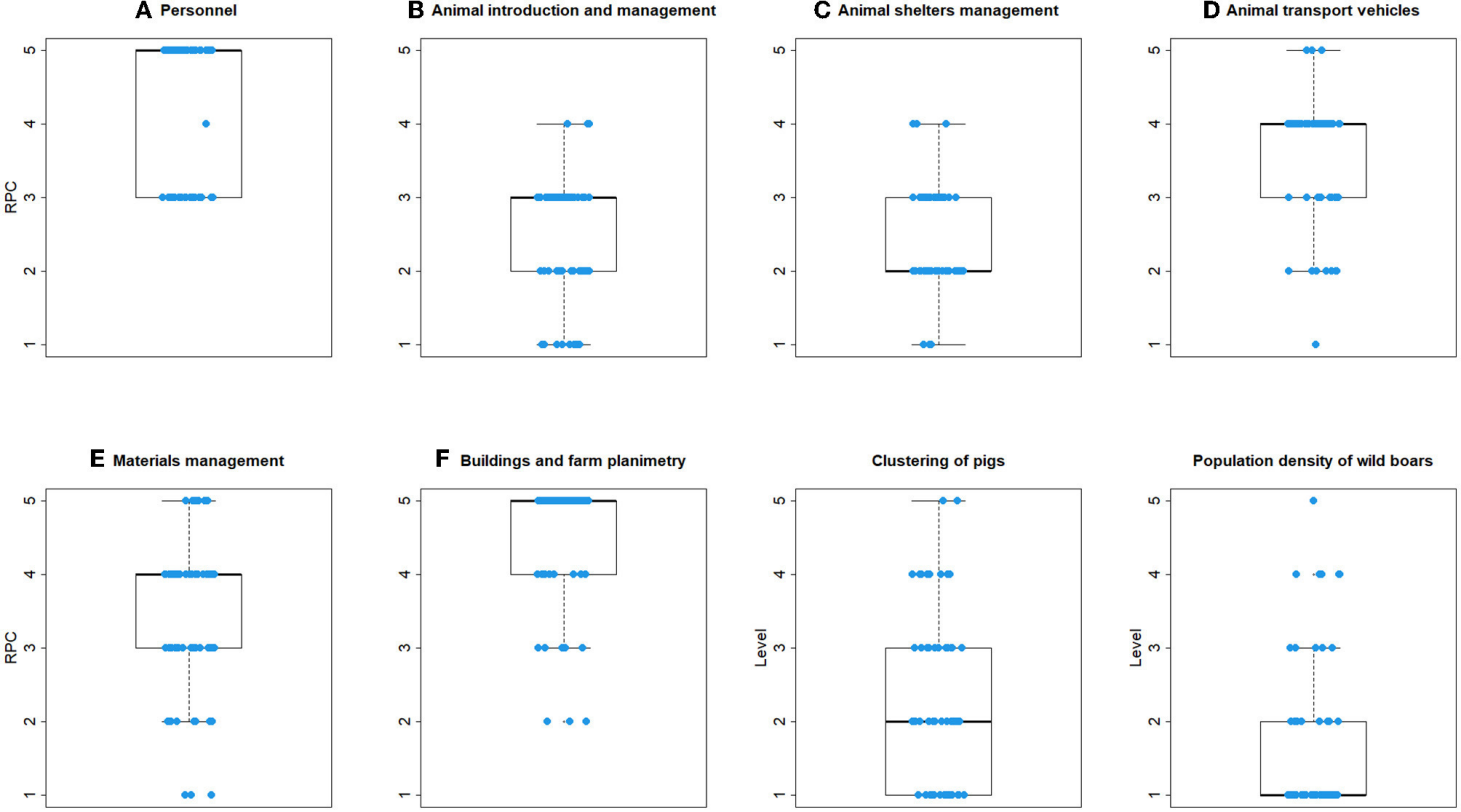
Boxplot of the distribution of risk priority codes (RPC) for each criterion, obtained by the application of FMEA, local density of pigs, and wild boar population density, estimated in 60 commercial pig farms in Italy. In the boxes, the thick horizontal line represents the median risk priority code; the base and the top of the boxes are the first, and third quartiles, respectively. Whiskers extend to a maximum of 1.5 times the interquartile range. Points represent extreme values. Blue circles represent individual farm's RPC's.

The distribution of RPC's resulting from the FMEA calculation in each individual commercial farm, considering the non-compliance and importance scores of the sub-criteria, are shown in Figure 4. The highest RPC score (median = 5) was obtained for biosecurity measures associated with personnel practices (main criterion A) and for buildings and farm planimetry (main criterion F). Median RPC = 4 was obtained for biosecurity measures associated with the management of animal transport vehicles (main criterion D) and with material management (i.e., feed, slurry, other vehicles; main criterion E). Lower, and upper limits of RPC's, which were obtained by adopting the corresponding 95% confidence limits of importance of sub-criteria in calculation, are shown in Supplementary Table S2. The effect of the variability of the importance was more limited for RPC's for the main criterion A, which were most consistently high.

**Figure 5:**
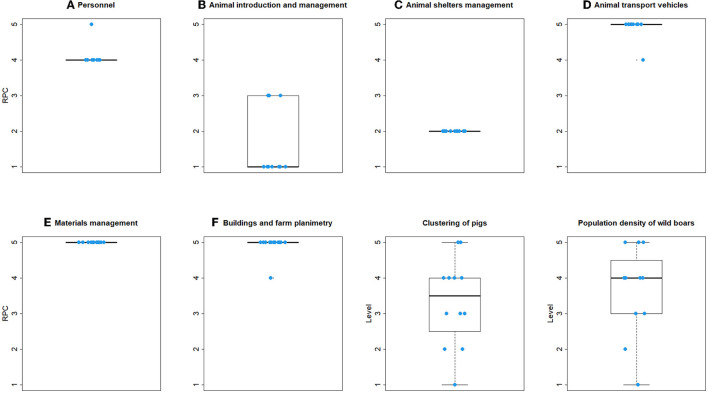
Boxplot of the distribution of risk priority codes (RPC) for each criterion, obtained by the application of FMEA, local density of pigs, and wild boar population density, estimated in 12 non-commercial and small commercial pig farms in Italy. In the boxes, the thick horizontal line represents the median risk priority code, whereas the base and the top of the boxes are the first, and third quartiles, respectively. Whiskers extend to a maximum of 1.5 times the interquartile range. Points represent extreme values. Blue circles represent individual farm's RPC's.

In non-commercial and small commercial farms, a median RPC of 5 was observed for criteria D, E, and F (animal transport vehicles; material management: feed, slurry, other vehicles, buildings, and farm planimetry). Criteria A showed median RPC = 4 (personnel). Better results were obtained for criteria C (animal shelter management), B (animal introduction and management) (Figure 5). Lower, and upper limits of RPC's (Supplementary Table S3) indicated some degree of variability, as the consequence of variable importance assignment, except for criteria B, and C.

### 3.4. Geographic risk factors

**Figure 6:**
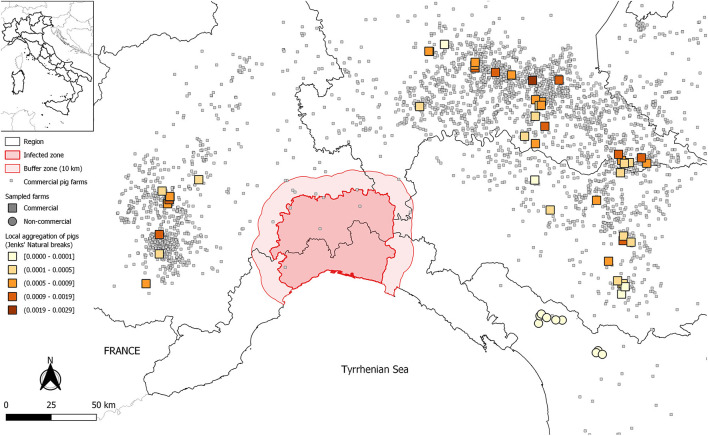
Geographic distribution of commercial pig farms in Italy, and of 72 visited farms, which were classified by G statistics, as an index of local density of farmed pigs. Commercial farms are identified by squares. Non-commercial and small commercial farms in north western Tuscany are identified by circles. Farm location colour is based on Jenks natural breaks classification method, of all farm types combined. The infected area for ASF (4) is reported.

Median wild boar density (first, third quartile) at the location of non-commercial and small commercial farms was 5.2 (4.1, 5.6) heads/km^2^, based upon 5 km resolution raster maps (33). It was greater than at locations of commercial farms, where it was 0.13 (0.02, 0.61) heads/km^2^ (Figure 1). On the other hand, the visited commercial farms in the Po River Valley were in densely populated livestock areas. The local density of pigs, as estimated by G statistics, was highest in Lombardy (Figure 6). The median (first, third quartile) number of farms, surrounding each commercial farm, within a 3 km distance, was 7.5 (3.75, 12.0), whereas the number of pigs was 13,234 (5,889, 28,216). Spatial density was very low for non-commercial/small commercial farms, with 1.0 (0.0, 2.0) farm, and 11.0 (0.0, 37.8) pigs, within 3 km from each farm.

### 3.5. Overall ranking for the risk of introduction of ASFV in pig farms

The ranking of commercial and of non-commercial/small commercial farms, in terms of the risk of ASFV introduction, based upon combination of on-farm criteria, population density of wild boars, and local density of domestic pigs are shown in Supplementary Tables S2, S3. Separate ranking of these farm types are presented, following the indications reported on DG SANTE working document.^8^ Among commercial farms, median (first, third quartile) rank was lowest in post-weaning units: 21.5 (9.2, 33.0), indicating a relatively high risk of ASFV introduction (Supplementary Table S2). Farms that were not specialized in a specific rearing phase (e.g., both post-weaning and fattening in the same farm), were characterised by a median rank of 29.0 (17.5, 50.5), whereas for fattening units: median = 30.0 (13.0, 42.0). Breeding units, median rank = 51.5 (31.8, 57.8), were at a relatively low risk of ASFV introduction. However, the observed differences among median ranks in different production phases, in commercial farms, was not statistically significant at the 0.05 significance level (*P*-value = 0.07). A weak, negative non-parametric correlation between risk rank and herd size of commercial farms, indicating a slight increase in risk with increasing herd size of commercial farms, was not statistically significant (Kendall's τ = −0.13, *p* = 0.14).

## 4. Discussion

This study was planned in the context of a larger research program on ASF (Defend European project), and focused on the development of a semi-quantitative risk assessment tool to estimate the biosecurity level of pig farms in a standardized and reproducible manner. The scoring system includes the most relevant aspects of biosecurity, which are specifically connected to ASF prevention and control. The aim was not to replace the other existing tools for the assessment of farm biosecurity (e.g., Biocheck.UGent™ or ClassyFarm), but to address specific risk of ASFV introduction in pig farms. In contrast to questionnaires in which no weights are given to the different measures and only the number of compliant items is considered, the modified FMEA includes an importance score, which was based upon opinions by a panel of expert (37). In our approach, the most dangerous failure mode of a biosecurity criterion is obtained by the highest evaluation of non-compliance on the most important sub-criterion (29). *Via* this process, the modified FMEA identifies weak points in biosecurity, and sets the basis to prioritize intervention on those specific biosecurity measures in individual farms, which are associated with high RPC's. Furthermore, in FMEA we combined data of different types and sources, taking into account the ordinal properties of five-level scales. By integrating RPC's (as obtained by FMEA of data collected in visited farms) and geographic risk factors (population density of wild boars and local density of farmed pigs), we obtained an overall ranking of pig farms, in terms of the risk of introduction of ASFV. Based upon such a transparent and easily communicable process, surveillance and intervention resources can be primarily dedicated to farm categories, production phases and geographical areas where the risk of infection is greatest (38).

In our approach, risk-ranking of pig farms corresponded to a decreasing ordering of counts of high RPC's and scores of geographical risk factors. In this way, farms with the highest frequency of scores = 5 were considered at greatest risk. According to this approach, farms with high non-compliance levels to important biosecurity components were considered at the greatest risk of ASFV introduction. An alternative approach could be adopted by ranking farms based on the overall sum of RPC's and geographical risk factors. However, in this way, the same sum could be obtained by different combinations of results, and more farms would be assigned the same risk rank, with a lesser weight of non-compliance to the most important sub-criteria.

It is important to highlight that the present study was entirely performed before the ASF occurrence in northern Italy. Indeed, several authors have suggested that the risk perception of a disease and its consequences on the farm is the main factor leading to the application of biosecurity measures. The greater application of biosecurity measures has been observed after outbreaks of diseases such as porcine reproductive and respiratory syndrome or influenza, as well as in densely populated areas of pigs, probably due to a higher perception of the transmission risk between neighbours (39, 40).

In the ranking of the sampled commercial farms, which we obtained by the application of FMEA and of geographical risk factors, the risk of ASFV introduction was lower in breeding herds than in other production phases, although difference among median ranks was not statistically significant at the 0.05 level. Silva et al. (14) also found that breeding herds were the ones with best biosecurity scores, since these were most likely to undergo certification and annual monitoring by the official veterinary service. Moreover, breeding farms are the top of the sanitary pyramid in pig production, they have a high sanitary status and a reduced risk of introduction of pathogens (41). Herd size was not correlated with risk in our sample of 60 commercial farms. Although most of the ASF outbreaks in Europe have occurred in small pig holdings (1), it should also be noted that smaller commercial farms require lower investment to implement biosecurity measures (14).

In the ranking of commercial farms, the first seven positions (i.e., the farms with highest risk of introduction of ASFV) were occupied by farms having RPC's = 5 in the main criteria A (personnel practices) and F (buildings and farm planimetry). Such criteria are under the direct responsibility of farmers, and they are influenced by the farm manager's decisions and investments. On the other hand, exposure to geographic risk factors, such as local density of farmed pigs, which is greatest in the main pig production areas, and wild boar population density, is beyond control by individual farmers, and it should be the object of national or regional disease prevention programs.

RPC = 5 for criterion A can be explained by the presence of several critical items. In fact, the experts selected 9 of the 98 items as *conditio sine qua non* to achieve a sufficient biosecurity level in the farm, and four of these were included in criterion A. In 63.3% of commercial farms, employees were involved in activities in other pig farms, often belonging to the same ownership. Personnel working on the same farm most of the time, occasionally worked in different farms to carry out tasks, such as loading/unloading live animals, or cleaning and disinfecting procedures. Furthermore, priorities for intervention were identified in 70% of the farms, which were characterised by improper access area, with overlapping clean and dirty areas. A shower was mandatory before entering only in 10% of farms, and a Danish entry was present in just 6–7% of farms. The combination of these non-compliances amplifies the seriousness of the risk of introducing the disease and provides clear indications on the priorities of intervention (42). The risk related to the personnel in the visited farms can be worsened by another priority intervention: the lack of specific training on biosecurity and the risk of introducing ASF, observed in 71.7% of the farms. Some authors reported that, in breeders' view, training on biosecurity, is not useful because often it is not well understood or adequately explained, despite it is a low-cost intervention (43). The results of the modified-FMEA also suggest the need for training on practices regarding the introduction and the consumption of food in the farm, as 36.7% of the establishments did not provide any specific indication. Indeed, waste food has frequently been implicated in the spread of ASFV (44, 45). However, swill feeding was not performed in any of the visited commercial farms, while it was carried out in two of 12 non-commercial and small commercial farms. Based on the work of Olševskis et al. (46), swill feeding is one of the most likely routes of transmission of ASFV to domestic pig farms, and it was selected among the critical items by the experts' panel in the present study. Our results identified a better situation in comparison with that reported by Boklund et al. (26) from Romania, a country which is known for having thousands of backyard farms and for using swill feeding as one of the few countries in Europe: the authors found that several farms fed swill to pigs (especially backyard farms), despite its total ban in the EU since 2002 (47).

The main criterion of building and farm planimetry (F) was identified as another priority for intervention in both commercial and non-commercial/small commercial farms. In particular, non-compliance in 70.0% of commercial farms and 100.0% of non-commercial/small commercial farms emerged in relation to the critical item of fencing, which were often incomplete. A perimeter fence with a permanently closed door that can only be opened from inside the farm was suggested by Alarcón et al. (48) as the crucial requirement for an efficient division between “inside” and “outside” the farm. Fences have also been tackled in the recent EFSA report on ASF in outdoor farms (49): the authors were 66–90% certain that if single solid or double fences were fully and properly implemented, in all outdoor pig farms in ASF affected areas of the EU, this would reduce the number of new outbreaks within a year by more than 50%, without requiring any other outdoor-specific control measures. Moreover, our prioritization indicated that around the farming area of 71.7% of commercial farms, an asphalted zone was missing. Debris and grass around the barns are considered as a risk because they allow the breeding of insects and rodents as a vehicle of infection, and attract wild animals (50). Domestic and wild animals were present in 60.0% of commercial farms. Furthermore, 85.0% of the farmers declared that they self-managed the rodent control plan (i.e., no external professional rodent control company was involved), and no farms had insect and bird control plans in place. Moreover, biosecurity practices during the transport of live animals through vehicles and their loading and unloading were not optimal. Notably, the vehicles used to transport animals between farms, slaughterhouses, and drivers can play an important role in the transmission of pathogens, as described by Alarcón et al. (48).

As expected, non-compliance with biosecurity measures was frequent in non-commercial and small commercial farms, where three critical items were never satisfied, and other three were only rarely satisfied, out of seven for which information was collected. Staff always had contact with other pig farms, external fences were absent, and no disinfectant was available. Procedures related to material management (feed, slurry, other vehicles; main criterion E) were also unsatisfactory. The results obtained in non-commercial/small commercial farms are in agreement with the EFSA report published in 2021 (51), which described small-scale farms as often characterised by little, if any, investment in farm infrastructure and equipment. In the literature, non-commercial farms are described as one of the weakest parts of the biosecurity chain and the biggest risk factor for ASF introduction in domestic pig populations (46, 52). Although non-commercial farms can be a dead-end in terms of the disease spread, units that sell animals at the local or regional levels can play a role in the spread of diseases (1). As a consequence, these farms must adopt the necessary control measures to mitigate the risk. On the small sample of non-commercial/small commercial farms, we applied a reduced version of our checklist, and the obtained risk ranking is not directly comparable with that of the 60 commercial farms. Nevertheless, the inclusion of these results in the present study is important due to the location of farms on the northern Apennines, where an ASF-infected area has been identified at ~ 150 km distance, and where the risk of ASFV diffusion by abundant wild boars was particularly high. These preliminary results set the basis for further larger scale studies on non-commercial, and outdoor pig farms in Apennine areas.

The implementation of certain biosecurity measure in farms requires considerable economical investments (e.g., building a proper perimetral fence around the farm). Conversely, non-compliance with other measures, which was observed in the present study, could not be attributed to economic constraints, but it was most likely due to established habits, and to a negative attitude toward biosecurity practices, which might be considered as time-consuming and not perceived as useful by the farmers (53). In fact, training of farm personnel, and good communication among all stakeholders can play a central role in ASF prevention. Worth mentioning that with the introduction of EU legislation 2021/605 (9), swine farmers are required to put into place a biosecurity plan against ASF. In this context, the information of farmers on weak points in biosecurity and on preventive measures may enhance their proactive role in the fight against ASF.

We selected commercial farms with the collaboration of a limited number of veterinarians and based upon the voluntary inclusion of farm owners. Results could, therefore, be affected by a bias. The application of our method to representative samples of farms would allow drawing more solid conclusions at the populations level. Further studies might be needed to validate this approach, possibly by evaluation of reproducibility of scoring on the same farms by multiple assessors. Moreover, the integration with up-to-date information on animal movements among farms, as described in Bellini et al. (54) would be useful for network-modelling the spread of the disease.

Results of FMEA were affected by choices in assigning non-compliance scores. Furthermore, scores of several sub-criteria were arbitrarily grouped into main criteria. Importance was assigned, to each sub-criterion, by a limited number of experts, and the variability of estimates, as expressed by 95% confidence limits, could affect RPC's of the main criteria (Supplementary Tables S2, S3). Such an effect was reduced for criterion A, for which non-compliance was generally high; this was in part due to the presence of critical items which, if not satisfied, invariably led to non-compliance score = 5. It is, therefore, evident that choices in assigning non-compliance and importance scores, and the selection of critical items can affect FMEA results. This must be taken into account when applying the method to different pig farming systems. In the case of geographic risk factors, different methods of classification into five ordered exposure levels (e.g., Jenks natural breaks vs. quintiles) also affect the overall ranking of pig farms. Moreover, boundaries of exposure levels were relative to the examined sample of farms, and will change when a different sample is assessed, especially if in areas with different wild boar and domestic pig densities. Ranking of farms in term of the risk of ASFV introduction must, therefore, be referred to the population at hand, and generalization should be considered with caution.

The adoption of our semi-quantitative risk assessment method might be useful to identify farms eligible to be part of a compartment. The compartment, following indications provided by the WOAH, is one or more establishments, separated from other susceptible populations by a common biosecurity management system, and with a specific animal health status with respect to one or more infections or infestations, for which the necessary surveillance, biosecurity, and control measures have been applied for the purposes of international trade or disease prevention and control in a country or zone. It is worth mentioning that the compartment concept was initially developed by the WOAH. However, recently, the possibility of implementing a compartment has also been established under the Animal Health Law, which means that compartmentalisation is now a disease control option applicable to the European Union.

## Data availability statement

The original contributions presented in the study are included in the article/Supplementary material, further inquiries can be directed to the corresponding author.

## Author contributions

AM and SB conceived of and designed the study. AS, FV, AR, GF, and AB designed the checklist and collected and compiled the data. GF, AM, and AB performed statistical analyses. AS, FV, GF, AR, SC, VC, AB, AM, GA, and SB drafted and edited the manuscript. All authors have contributed to the manuscript and approved the submitted version.

## Footnotes

^1^https://storymaps.arcgis.com/stories/7f16f51731654a4ea7ec54d6bc1f90d4, accessed on 02 January 2023.

^2^www.classyfarm.it, accessed on 08 November 2022.

^3^available at www.apiq.com.au, accessed on 24 November 2022.

^4^available at www.risikoampel.uni-vechta.de, accessed on 24 November 2022.

^5^www.classyfarm.it, accessed on 08 November 2022.

^6^Directorate General for Health and Food Safety Strategic Approach to the Management of African Swine Fever for the EU 2020. Available online: https://ec.europa.eu/food/sites/food/files/animals/docs/ad_control-measures_asf_wrk-doc-sante-2015-7113.pdf (accessed on 24 November 2022).

^7^VetInfo, Italian National Zootechnical Registry, updated 31 December 2021; https://www.vetinfo.it/j6_statistiche/#/report-pbi/31, accessed on 02 October 2022.

^8^DG SANTE, Directorate General for Health and Food Safety. Strategic Approach to the Management of African Swine Fever for the EU. 2015. SANTE/7113/2015-Rev 12. Available online: https://ec.europa.eu/food/sites/food/files/animals/docs/ad_control-measures_asf_wrk-doc-sante-2015-7113.pdf (accessed on 27 September 2022).
