# Supplementary material for: Semi-quantitative risk assessment of African swine fever virus introduction in pig farms
Source: Front Vet Sci. 2023 Jan 27;10:1017001. doi: 10.3389/fvets.2023.1017001 (PMC9911915; doi:10.3389/fvets.2023.1017001)
Supplement: Supplementary file 1 [file Table_1.docx]

Supplementary Material

Semi-quantitative risk assessment of African swine fever virus introduction in pig farms

A. Scollo*, F. Valentini, G. Franceschini, A. Rusinà, S. Calò, V. Cappa, A. Bellato, A. Mannelli, G. L. Alborali, S. Bellini

*** Correspondence:** A. Scollo: annalisa.scollo@unito.it

**Supplementary table 1.** Complete list of the 98 items considered in the checklist, and non-compliance (negative answer to dichotomous answers) in 60 commercial farms, and in 12 non-commercial (including small commercial farms; ≤ 20 heads). Empty cells for non-commercial farms indicate that information on the corresponding items was not collected.

*items that required the selection of one among 5 mutually exclusive options, of increasing non-compliance level (from 1, to 5) with optimal biosecurity practices.

|  |  |  |  | % of non-compliant farms | |
| --- | --- | --- | --- | --- | --- |
| Criterion | Item id | Sub-criterion | Item description | Commercial | Non-commercial and small commercial |
| A: personnel | 1 | A1 Entrance of personnel into the farm | Change of clothes and footwear is carried out | 3.3 | 91.7 |
|  | 2 |  | Clothing and footwear are cleaned and disinfected regularly or are disposable. | 20.0 | 100.0 |
|  | 3 |  | There is a locker room with toilets and cleaners and a clear separation between clean and dirty areas: the entrance to the locker room (dirty) is DIFFERENT from the exit (clean), so that there are no areas of overlapping dirty-clean transit | 70.0 | 100.0 |
|  | 4 |  | There is a Danish entry, i.e. a bench that totally separates the dirty and clean areas and remember the personnel the threshold | 93.3 | 16.7 |
|  | 5 |  | Personnel take a shower before entering the farm | 90.0 | 100.0 |
|  | 6 | A2 Contact of personnel with other pigs and wild boar hunting | The staff has no other pigs | 23.3 | 83.3 |
|  | 7 |  | Staff has no contact with other pig holdings | 63.3 | 100.0 |
|  | 8 |  | Staff do not engage in wild boar hunting activities | 10.0 | 83.3 |
|  | 9 |  | If it comes into contact with animals from other farms or suids (wild boars), the staff should rest for at least 48 h before entering the farm after this activity. | 75.5 | 100.0 |
|  | 10* | A3 Food introduction by personnel | There is a ban on staff bringing in their own food, or there is an in-house canteen. | 35.0 | 0.0 |
|  |  |  | Staff may bring in their own food, but it may only be consumed in a defined area, the area is physically separated from the animal holding area, and staff may only consume food during designated breaks | 18.3 | 91.7 |
|  |  |  | Staff may bring in their own food, but it may only be consumed in a defined area, the area is physically separated from the animal holding area, staff may consume the food at any time during the day | 5.0 | 8.3 |
|  |  |  | Staff may bring in their own food, but it may only be consumed in a defined area, the area is not physically separated from the animal holding area, staff may consume food at any time of the day or during established breaks | 5.0 | 0.0 |
|  |  |  | Staff may bring in food, and there is no indication as to the area in which it must or may be consumed | 36.7 | 0.0 |
|  | 11* | A4 Personnel training | All personnel working on the farm (workers, technicians, managers, keepers/owners) are adequately trained on biosecurity and the risks of intro ASF virus and the course is less than a year old (documentation) | 15.0 | 0.0 |
|  |  |  | All personnel working on the farm (workers, technicians, managers, keepers/owners) are adequately trained on biosecurity and the risks of intro ASF virus and the course is more than a year old | 13.3 | 0.0 |
|  |  |  | Only a portion of the staff working on the farm are trained in biosecurity and the risks of introducing ASF, and the course is less than a year old | 30.0 | 0.0 |
|  |  |  | Only a portion of the staff working on the farm are trained in biosecurity and the risks of introducing ASF, and the course is more than a year old | 13.3 | 0.0 |
|  |  |  | The farmer and staff are either not trained at all on biosecurity and the risks of introducing ASF or there is no clear evidence of courses | 28.3 | 100.0 |
| B: Animal introduction and management | 12 | B1 Health/feeding/breeding status of introduced pigs | The health status of all animals for ASF is known, prior to their entry into the herd | 0.0 | 0.0 |
|  | 13 |  | All animals are correctly identified on the farm | 0.0 | 0.0 |
|  | 14 |  | There is an accurate and punctual registration of movements both in/out and within the holding structures | 0.0 | 83.3 |
|  | 15 |  | The seminal material comes from authorized semen collection centers or the reassembly is carried out by in-house breeders or, in the case of natural service, the boars have undergone the appropriate diagnostic tests. | 9.1 | 100.0 |
|  | 16 |  | Animals are not fed catering waste, canteen waste or household leftovers | 0.0 | 16.7 |
|  | 17 |  | Is family slaughter, if it occurs, done under the supervision of a veterinarian? (Documentation) Only for family farms | 13.3 | 0.0 |
|  | 18* | B2 Number of farms of origin of the introduced pigs | The farm introduces animals throughout the year from one or two farms | 65.0 | 100.0 |
|  |  |  | The farm introduces animals throughout the year from three farms | 11.7 | 0.0 |
|  |  |  | The farm introduces animals throughout the year from four farms | 16.7 | 0.0 |
|  |  |  | The farm introduces animals during the year from five farms | 3.3 | 0.0 |
|  |  |  | The farm introduces animals during the year from more than five farms | 3.3 | 0.0 |
|  | 19* | B3 Management of animals with an impaired growth | There are no "scraps" (animals with impaired growth) they are all euthanized and the vehicle collects the scraps from a single collection point | 16.7 | 100.0 |
|  |  |  | Scraps are taken away at the end of the cycle and the vehicle collects them from a single collection point | 30.0 | 0.0 |
|  |  |  | Scraps are taken away at the end of the cycle and the vehicle does not collect it from a single collection point | 1.7 | 0.0 |
|  |  |  | Scraps are taken away during the cycle and the vehicle collects them from a single collection point | 45.0 | 0.0 |
|  |  |  | Scraps are taken away during the cycle and the vehicle does not collect them from a single collection point | 6.7 | 0.0 |
| C: Animal shelters management | 20 | C1 Quarantine | A quarantine of at least 30 days is carried out (with animals serologically tested and visited) | 55.0 |  |
|  | 21 |  | There is a physically separate dedicated premise for quarantine | 10.0 |  |
|  | 22 |  | Biosecurity measures for staff access to the quarantine area are implemented | 60.0 |  |
|  | 23 |  | Quarantine premises have separate entrances | 30.0 |  |
|  | 24 |  | Quarantine premises have separate pits | 50.0 |  |
|  | 25 |  | All-in/all-out and a suitable sanitary break period is practiced for the quarantine premises | 20.0 |  |
|  | 26 |  | For quarantine management, staff change clothes entirely and use dedicated equipment | 40.0 |  |
|  | 27 | C2 Internal animal flow and cleaning and disinfection procedures | Management of all-in/all-out: it is complete, no temporal overlapping of batches in the whole farm or quarantine is carried out | 18.6 |  |
|  | 28 |  | At least 5 days of sanitary break between emptying and introducing the new animals is followed | 18.3 |  |
|  | 29 |  | Structures are meticulously washed using disinfectants between batches | 1.7 |  |
|  | 30 |  | Pressure equipment is available for cleaning, washing and disinfection | 0.0 |  |
|  | 31 | C3 Vaccine prophylaxis and treatments for other infectious diseases | There is a hygienic and sanitary procedure/protocol of handling equipment used for vaccine prophylaxis and therapeutic treatments | 55.0 | 100.0 |
|  | 32 |  | Any results of official or self-inspection analyses are present (samples taken from animals or other matrices) | 0.0 | 100.0 |
|  | 33 |  | There is a documented vaccination plan | 8.3 | 25.0 |
|  | 34 |  | There is a system for recording company health, breeding, reproduction and production data | 0.0 | 75.0 |
|  | 35 | C4 Structure and buildings | The farm has physical or natural barriers that circumscribe the animal housing and management area | 33.3 | 0.0 |
|  | 36 |  | The area of housing and management of animals, has a wall or a fence suitable to prevent the entry of other animals including wild ones | 50.0 | 0.0 |
|  | 37 |  | There is a planimetry, with sheds and stalls univocally numbered, through which it is possible to verify the unidirectional flow of the movements of the animals in the farm and identify the groups of animals. | 3.3 | 100.0 |
|  | 38 |  | Animals do not have the ability to access/live in the outdoor environment, or animals do have the ability to access only an external dunging area totally confined by a solid wall 1.5 m high. | 10.0 | 0.0 |
|  | 39 |  | Livestock housing shall have airtight, well-maintained, seamless walls, floors, and windows and doors that can be effectively cleaned and disinfected | 31.7 | 0.0 |
|  | 40 | C5 Dead pigs’ management | Where piglet carcasses are temporarily stored on the farm premises, pending their removal, the containers used shall be properly sealed and suitable for storage | 52.9 |  |
|  | 41 |  | Dead animal carcasses shall be removed from farm premises within 24 hours of death. | 11.7 |  |
|  | 42 |  | Dead animal carcasses shall be stored in a suitable, functioning insulated container or in a sealed cold storage room for disposal in accordance with health regulations | 23.3 |  |
|  | 43 |  | The area underneath the carcass container/cold storage room, is suitable both for the collection of any leaking materials or liquids and for cleaning and disinfection | 77.4 |  |
|  | 44 |  | The area underneath of the carcass container/cold storage room, is suitable for cleaning and disinfection | 41.5 |  |
| D: Animal transport vehicles | 45 | D1 Live animal transport vehicles | There is an area for the disinfection of live animal transport vehicles located near the access to the farm but separate from the area designated for housing. | 62.7 | 100.0 |
|  | 46 |  | No live animal transport vehicles enter the farm's clean area | 55.0 | 100.0 |
|  | 47 |  | All farm vehicles do not have access to other pig farms | 84.7 | 0.0 |
|  | 48 |  | Vehicles are cleaned and disinfected (including wheels and fenders) with disinfectants proven effective for ASF before entering the clean area | 37.3 | 100.0 |
|  | 49 | D2 Live animal unloading/loading | There is a loading bay for loading and unloading animals | 75.0 | 8.3 |
|  | 50 |  | Specific gates are in place to prevent animals from turning back | 69.5 | 100.0 |
|  | 51 |  | The unloading/loading of live animals takes place outside the farm's clean area | 55.0 | 91.7 |
|  | 52 |  | The unloading/loading of live animals takes place with a single-load vehicle. | 0.0 | 100.0 |
|  | 53 | D3 Carcasses disposal | The carcass container/cold storage rooms are outside the clean area of the farm | 32.7 |  |
|  | 54 |  | Emptying the carcass container/storage room takes place by non-contact tipping directly into the carcass removal truck | 25.9 |  |
|  | 55 |  | The contents of the carcass container/cold storage rooms are transported by staff to the outside of the farm | 42.3 |  |
|  | 56 |  | The carcass removal truck does not enter the farm | 50.0 |  |
|  | 57 | D4 Equipment and tools for loading/unloading live animals | While loading animals, transporters help inside the truck but never enter any clean area of the farm, which is clearly demarcated | 3.3 |  |
|  | 58 |  | Clothing provided to transporters is company or freshly laundered, and boots are company issued | 11.7 |  |
|  | 59 |  | The loading bay and the tools used are never switched from the dirty to the clean area and vice versa | 41.7 |  |
|  | 60 |  | The loading bay and the tools used are disinfected after each use | 71.7 |  |
|  | 61 |  | The weighbridge for the live animal vehicles, if any, is located outside the animal housing and governing area | 12.2 |  |
| E: Material management: feed and fodder, slurry, other vehicles | 62 | E1 Procedures for loading/unloading of feed and materials | The unloading of feed, bedding, outdoor material, etc. takes place outside the perimeter of the clean area. | 46.7 |  |
|  | 63 |  | Vehicles and outside operators do not stop or pass through a clean area to reach silos/barn/depot | 43.3 |  |
|  | 64 |  | If for the retrieval of the materials to be brought in, company operators must step on a common unloading area with external operators or pass through a dirty area, they use dedicated clothing and footwear | 90.0 |  |
|  | 65 |  | For the collection of the material to be brought in, company operators use dedicated footwear and clothing | 79.7 |  |
|  | 66 |  | The truck route is such that it crosses the operators' routes, but the operators adopt procedures to remedy the problem or the truck route does not cross the operators' routes | 61.7 |  |
|  | 67 |  | The silos and the hayloft are inside the farm perimeter but not in contact with animals and/or operator routes (e.g. the staff adopts procedures to avoid the problem such as the change of footwear and clothes if they cross the path of vehicles or external operators) or the silos and the hayloft are outside the farm perimeter | 55.0 |  |
|  | 68 | E2 Feed and materials storage | The external material (bedding, arching material...) is stored for 30 days or treated with UV rays. | 81.4 |  |
|  | 69 |  | Forage material is stored for 30 days or treated with UV light | 81.4 |  |
|  | 70 |  | The material is stored in closed and covered premises | 28.8 |  |
|  | 71 |  | Feed storage is protected from animals by sparrow nets/sealing/covering | 20.3 |  |
|  | 72 | E3 Slurry management | The slurry tank is located outside the perimeter of the clean area | 28.3 |  |
|  | 73 |  | The slurry tank can only be reached by a route outside the perimeter of the clean area of the farm | 51.7 |  |
|  | 74 |  | The slurry tank is not accessible to wild animals | 44.8 |  |
|  | 75 |  | There are entrances for slurry transport operations differentiated from those of the animal housing/management area | 65.5 |  |
|  | 76 |  | Slurry of other origin are not spread in the fields adjacent to the farm | 15.0 |  |
|  | 77 | E4 Vehicles for loading/unloading feed and materials | Vehicles other than those for loading/unloading live animals and carcasses (transport of feed, materials, owned vehicles) do not enter the farm's clean area. | 51.7 | 100.0 |
|  | 78 |  | The vehicles are disinfected in advance on a dedicated yard and there is documentation | 54.5 | 100.0 |
|  | 79 |  | Vehicles neither transit nor are parked anywhere on the farm | 29.8 | 8.3 |
|  | 80 |  | Vehicles do not come into contact with the area where animals are present (clean area) | 17.5 | 100.0 |
| F: Buildings and farm planimetry | 81 | F1 Farm perimeter barriers | There is an external fence for the entire farm perimeter that prevents the entrance of wild animals and visitors | 70.0 | 100.0 |
|  | 82 |  | Access points are closed by a gate | 21.7 | 0.0 |
|  | 83 |  | Signs prohibiting access for unauthorized persons are present and clearly visible at the entrance | 21.7 | 100.0 |
|  | 84 |  | The farm has a dedicated area, located before the entrance barrier, for the parking of staff vehicles. | 53.3 | 0.0 |
|  | 85 |  | The pavement around the farm is asphalted | 71.7 | 100.0 |
|  | 86 |  | There is no unused or waste material around the farm, the farm is well maintained, there are no bushes, brushwood around the farm | 60.0 | 100.0 |
|  | 87 | F2 Other animals and cleaning and disinfection procedures | No pets are present and have access outside the farm perimeter | 60.0 | 100.0 |
|  | 88 |  | No other livestock species are farmed | 5.0 | 100.0 |
|  | 89 |  | Disinfectants with proven efficacy against ASF are available | 0.0 | 100.0 |
|  | 90 |  | There is a procedure/protocol of cleaning and disinfection of housing facilities and equipment after the end of each production cycle. | 25.0 | 100.0 |
|  | 91 | F3 Pest and rodent control | Rodent and pest control are checked at least every <6 months and there is documentation | 67.0 | 83.3 |
|  | 92 |  | Rodent control is carried out by an external specialized company | 58.3 | 25.0 |
|  | 93 |  | Pest control is carried out by an external specialized company | 85.0 | 91.7 |
|  | 94 |  | Sparrow nets are in placed or it is otherwise ensured that birds cannot enter the premises | 78.3 | 100.0 |
|  | 95 | F4 Visitors | The farm has a designated area, located before the entrance barrier, for visitors' vehicles to park | 81.7 | 100.0 |
|  | 96 |  | The farm has a dedicated filter area used as an hygiene lock for visitors | 66.7 | 100.0 |
|  | 97 |  | Rooms used as visitors' hygiene lock are adequately equipped with toilets and cleaners | 5.0 | 25.0 |
|  | 98 |  | There is an up-to-date visitors' logbook indicating at least the date, first and last name of the visitor, reason for the visit and vehicle registration number | 63.3 | 100.0 |

**Supplementary table 2**. Ranking of the 60 commercial farms in terms of the risk of ASFV introduction based upon combination of on-farm criteria, population density of wild boars, and local clustering of domestic pig. RPC: risk priority code. Limits: values of RPC’s, which were obtained by using the 95% confidence limits of importance scores assigned to sub-criteria. Production type: breeding; post-weaning - from weaning to approximately 30 kg of body weight; fattening – from approximately 30 kg of body weight to slaughter; not specialized (NS) – more than one rearing phase on the same farm. Herd size: in case of post-weaning and fattening sites: number of farmed pigs present the day of the visit; in case of breeding farms: number of productive sows present the day of the visit.

|  | **Biosecurity criteria** RPC’s (limits) | | | | | |  |  |  |  |
| --- | --- | --- | --- | --- | --- | --- | --- | --- | --- | --- |
| Rank | A | B | C | D | E | F | Level of pig clustering | Level of wild boar density | Prod. type | Herd size |
| 1 | 5 (5, 5) | 3 (3, 4) | 4 (4, 4) | 4 (3, 5) | 5 (4, 5) | 5 (4, 5) | 1 | 1 | Fattening | 1150 |
| 1 | 5 (5, 5) | 3 (3, 4) | 4 (4, 4) | 4 (3, 4) | 5 (4, 5) | 5 (4, 5) | 1 | 1 | Fattening | 1999 |
| 3 | 5 (5, 5) | 3 (3, 4) | 3 (3, 3) | 4 (3, 4) | 5 (4, 5) | 5 (4, 5) | 2 | 1 | Fattening | 2600 |
| 3 | 5 (5, 5) | 2 (2, 2) | 3 (3, 5) | 4 (3, 4) | 5 (4, 5) | 5 (4, 5) | 3 | 1 | Weaning | 5100 |
| 5 | 5 (5, 5) | 3 (3, 4) | 3 (3, 3) | 4 (3, 4) | 4 (3, 4) | 5 (4, 5) | 4 | 1 | NS | 9145 |
| 6 | 5 (5, 5) | 4 (3, 5) | 4 (4, 5) | 3 (3, 4) | 4 (4, 5) | 5 (4, 5) | 2 | 2 | Fattening | 8000 |
| 7 | 5 (5, 5) | 4 (3, 4) | 3 (3, 3) | 4 (3, 4) | 4 (3, 5) | 5 (4, 5) | 1 | 1 | Fattening | 1130 |
| 8 | 5 (5, 5) | 3 (3, 4) | 3 (3, 3) | 4 (3, 5) | 5 (4, 5) | 4 (3, 4) | 1 | 3 | Fattening | 1250 |
| 8 | 5 (5, 5) | 3 (3, 4) | 3 (3, 3) | 4 (3, 5) | 4 (4, 5) | 5 (4, 5) | 3 | 1 | NS | 702 |
| 8 | 3 (3, 5) | 3 (3, 4) | 3 (3, 3) | 4 (3, 4) | 5 (4, 5) | 5 (4, 5) | 1 | 4 | Weaning | 11000 |
| 8 | 3 (3, 3) | 3 (3, 4) | 3 (3, 5) | 4 (3, 5) | 5 (4, 5) | 5 (4, 5) | 4 | 1 | Fattening | 5500 |
| 12 | 5 (5, 5) | 4 (3, 5) | 3 (3, 5) | 3 (3, 4) | 4 (4, 5) | 5 (4, 5) | 2 | 2 | Fattening | 1999 |
| 13 | 5 (5, 5) | 2 (2, 2) | 3 (3, 5) | 4 (3, 4) | 4 (3, 5) | 5 (4, 5) | 3 | 1 | Weaning | 2550 |
| 13 | 5 (5, 5) | 2 (2, 2) | 3 (3, 3) | 4 (3, 4) | 3 (2, 3) | 5 (4, 5) | 1 | 4 | Fattening | 380 |
| 13 | 5 (5, 5) | 3 (3, 4) | 2 (2, 2) | 3 (3, 4) | 4 (3, 4) | 5 (4, 5) | 4 | 1 | Fattening | 6000 |
| 13 | 5 (5, 5) | 3 (3, 4) | 3 (3, 3) | 4 (3, 5) | 4 (3, 5) | 5 (4, 5) | 2 | 1 | NS | 5802 |
| 17 | 5 (5, 5) | 3 (3, 4) | 3 (3, 5) | 4 (3, 4) | 4 (3, 4) | 5 (4, 5) | 1 | 1 | NS | 5148 |
| 18 | 5 (5, 5) | 2 (2, 2) | 2 (2, 3) | 4 (3, 4) | 3 (2, 3) | 5 (4, 5) | 1 | 4 | Fattening | 340 |
| 18 | 5 (5, 5) | 2 (2, 2) | 2 (2, 3) | 4 (3, 4) | 3 (2, 3) | 5 (4, 5) | 1 | 4 | Fattening | 950 |
| 18 | 5 (5, 5) | 2 (2, 2) | 3 (3, 3) | 4 (3, 4) | 4 (3, 5) | 5 (4, 5) | 1 | 2 | Fattening | 750 |
| 18 | 5 (5, 5) | 2 (2, 2) | 2 (2, 3) | 4 (3, 4) | 4 (4, 4) | 5 (4, 5) | 3 | 1 | NS | 5200 |
| 18 | 3 (3, 3) | 2 (2, 2) | 2 (2, 3) | 5 (3, 5) | 4 (4, 5) | 5 (4, 5) | 4 | 1 | Fattening | 1850 |
| 23 | 5 (5, 5) | 3 (3, 4) | 2 (2, 3) | 5 (3, 5) | 4 (3, 5) | 4 (3, 4) | 1 | 1 | Fattening | 1850 |
| 23 | 5 (5, 5) | 2 (2, 2) | 3 (3, 3) | 4 (3, 4) | 4 (3, 5) | 5 (4, 5) | 1 | 1 | Fattening | 1200 |
| 23 | 5 (5, 5) | 3 (3, 4) | 2 (2, 2) | 1 (1, 1) | 4 (3, 4) | 4 (3, 4) | 5 | 1 | Breeding | 2805 |
| 26 | 5 (5, 5) | 3 (3, 4) | 3 (3, 3) | 3 (3, 4) | 4 (3, 5) | 5 (4, 5) | 3 | 1 | NS | 4620 |
| 27 | 5 (5, 5) | 3 (3, 4) | 3 (3, 3) | 2 (2, 2) | 3 (2, 4) | 5 (4, 5) | 4 | 1 | Breeding | 2000 |
| 27 | 5 (5, 5) | 3 (3, 4) | 3 (3, 5) | 3 (3, 4) | 4 (3, 5) | 5 (4, 5) | 2 | 1 | NS | 6700 |
| 29 | 3 (3, 3) | 1 (1, 1) | 3 (3, 5) | 3 (3, 3) | 4 (3, 4) | 5 (4, 5) | 5 | 1 | NS | 42000 |
| 30 | 5 (5, 5) | 3 (3, 4) | 2 (2, 3) | 3 (3, 3) | 4 (3, 4) | 5 (4, 5) | 2 | 1 | Fattening | 1170 |
| 30 | 5 (5, 5) | 1 (1, 1) | 2 (2, 3) | 4 (3, 4) | 3 (3, 3) | 5 (4, 5) | 3 | 2 | Weaning | 3200 |
| 32 | 5 (5, 5) | 2 (2, 2) | 2 (2, 3) | 4 (3, 4) | 3 (3, 3) | 5 (4, 5) | 2 | 1 | Fattening | 1100 |
| 32 | 5 (5, 5) | 2 (2, 2) | 2 (2, 3) | 4 (3, 4) | 3 (2, 3) | 5 (4, 5) | 2 | 1 | Fattening | 500 |
| 34 | 5 (5, 5) | 1 (1, 1) | 2 (2, 3) | 4 (3, 4) | 3 (2, 3) | 5 (4, 5) | 2 | 1 | Weaning | 1600 |
| 35 | 5 (5, 5) | 2 (2, 2) | 2 (2, 2) | 2 (2, 2) | 3 (2, 5) | 5 (4, 5) | 2 | 1 | Fattening | 6500 |
| 36 | 5 (5, 5) | 1 (1, 1) | 2 (2, 2) | 2 (2, 2) | 2 (2, 2) | 5 (4, 5) | 2 | 2 | NS | 1800 |
| 37 | 4 (3, 4) | 4 (3, 4) | 2 (2, 3) | 4 (3, 4) | 4 (3, 5) | 5 (4, 5) | 4 | 1 | Fattening | 10000 |
| 38 | 5 (5, 5) | 1 (1, 1) | 2 (2, 3) | 4 (3, 4) | 4 (4, 5) | 4 (3, 4) | 3 | 1 | Weaning | 1600 |
| 39 | 5 (5, 5) | 1 (1, 1) | 2 (2, 3) | 4 (3, 4) | 4 (3, 4) | 4 (3, 4) | 1 | 2 | Fattening | 1720 |
| 40 | 5 (5, 5) | 1 (1, 1) | 2 (2, 3) | 4 (3, 4) | 4 (3, 5) | 4 (3, 4) | 1 | 1 | Fattening | 50 |
| 41 | 5 (5, 5) | 3 (3, 3) | 3 (2, 5) | 4 (3, 4) | 4 (3, 5) | 3 (2, 3) | 3 | 3 | Fattening | 4200 |
| 42 | 3 (3, 3) | 3 (3, 3) | 2 (2, 3) | 4 (3, 4) | 3 (3, 3) | 5 (4, 5) | 4 | 2 | Fattening | 2850 |
| 43 | 3 (3, 3) | 3 (3, 3) | 3 (3, 5) | 4 (3, 4) | 4 (3, 5) | 5 (4, 5) | 1 | 1 | Fattening | 2900 |
| 44 | 5 (5, 5) | 2 (2, 2) | 2 (2, 3) | 3 (3, 3) | 3 (2, 3) | 4 (3, 4) | 1 | 4 | Fattening | 835 |
| 45 | 5 (5, 5) | 1 (1, 1) | 2 (2, 3) | 4 (3, 4) | 4 (3, 5) | 3 (3, 3) | 1 | 1 | Fattening | 440 |
| 46 | 3 (3, 5) | 3 (3, 4) | 3 (3, 3) | 3 (3, 3) | 3 (3, 4) | 5 (4, 5) | 1 | 4 | Breeding | 524 |
| 47 | 3 (3, 3) | 3 (3, 3) | 2 (2, 2) | 4 (3, 4) | 3 (2, 3) | 5 (4, 5) | 3 | 1 | Fattening | 1800 |
| 48 | 3 (3, 5) | 3 (3, 4) | 2 (2, 3) | 4 (3, 5) | 2 (2, 4) | 3 (3, 3) | 2 | 5 | NS | 736 |
| 49 | 3 (3, 3) | 2 (2, 2) | 2 (2, 3) | 5 (3, 5) | 3 (3, 3) | 4 (3, 4) | 2 | 1 | Fattening | 1500 |
| 49 | 3 (3, 3) | 2 (2, 2) | 2 (2, 3) | 4 (3, 4) | 3 (3, 3) | 5 (4, 5) | 2 | 1 | Fattening | 2494 |
| 49 | 3 (3, 5) | 3 (3, 4) | 2 (2, 3) | 2 (2, 2) | 2 (2, 3) | 5 (4, 5) | 4 | 1 | NS | 450 |
| 52 | 3 (3, 5) | 3 (3, 4) | 2 (2, 3) | 2 (2, 2) | 2 (2, 3) | 5 (4, 5) | 3 | 3 | NS | 2680 |
| 53 | 3 (3, 5) | 3 (3, 4) | 2 (2, 2) | 2 (2, 2) | 1 (1, 1) | 5 (4, 5) | 1 | 3 | Fattening | 1980 |
| 54 | 3 (3, 5) | 3 (3, 4) | 2 (2, 2) | 2 (2, 2) | 1 (1, 1) | 5 (4, 5) | 2 | 1 | NS | 2596 |
| 55 | 3 (3, 5) | 3 (3, 4) | 3 (2, 5) | 4 (3, 4) | 4 (3, 5) | 4 (3, 4) | 4 | 1 | Fattening | 9180 |
| 56 | 3 (3, 5) | 3 (3, 4) | 2 (2, 3) | 4 (3, 5) | 2 (2, 4) | 3 (3, 3) | 3 | 1 | NS | 1680 |
| 57 | 3 (3, 5) | 3 (3, 4) | 1 (1, 1) | 2 (2, 2) | 2 (2, 3) | 2 (2, 2) | 4 | 1 | Breeding | 9582 |
| 58 | 3 (3, 5) | 3 (3, 4) | 1 (1, 1) | 2 (2, 2) | 2 (2, 3) | 2 (2, 2) | 3 | 3 | Breeding | 515 |
| 59 | 3 (2, 3) | 1 (1, 1) | 2 (2, 2) | 3 (3, 3) | 1 (1, 1) | 3 (2, 3) | 2 | 2 | Breeding | 1326 |
| 59 | 3 (3, 5) | 3 (3, 4) | 1 (1, 1) | 2 (2, 2) | 2 (2, 3) | 2 (2, 2) | 3 | 1 | NS | 660 |

**Supplementary table 3**. Ranking of 12 non-commercial and small commercial (≤ 21 heads) farms in north-western Tuscany, terms of the risk of ASFV introduction based upon combination of on-farm criteria, population density of wild boars, and local clustering of domestic pigs. RPC: risk priority code. Limits: values of RPC’s, which were obtained by using the 95% confidence limits of importance scores assigned to sub-criteria. Production type: not specialized (NS) – more than one rearing phase on the same farm. Herd size: in case of post-weaning and fattening sites - number of farmed pigs present the day of the visit; in case of breeding farms - number of productive sows present the day of the visit.

|  | Biosecurity criteria RPC’s (limits) | | | | | | |  | |  | |  | |  |
| --- | --- | --- | --- | --- | --- | --- | --- | --- | --- | --- | --- | --- | --- | --- |
| Rank | A | B | C | D | E | F | Level of pig clustering | | Level of wild boar density | | Production type | | Herd size | |
| 1 | 4 (3, 5) | 3 (3, 3) | 2 (2, 2) | 5 (3, 5) | 5 (4, 5) | 5 (4, 5) | 5 | | 5 | | NS | | 20 | |
| 2 | 4 (3, 5) | 3 (3, 3) | 2 (2, 2) | 5 (3, 5) | 5 (4, 5) | 5 (4, 5) | 4 | | 5 | | NS | | 15 | |
| 3 | 4 (3, 5) | 3 (3, 3) | 2 (2, 2) | 5 (3, 5) | 5 (4, 5) | 5 (4, 5) | 5 | | 2 | | NS | | 10 | |
| 4 | 5 (5, 5) | 1 (1, 1) | 2 (2, 2) | 5 (3, 5) | 5 (4, 5) | 5 (4, 5) | 3 | | 4 | | NS | | 2 | |
| 5 | 4 (3, 5) | 1 (1, 1) | 2 (2, 2) | 5 (3, 5) | 5 (4, 5) | 5 (4, 5) | 2 | | 5 | | NS | | 8 | |
| 6 | 4 (3, 5) | 1 (1, 1) | 2 (2, 2) | 5 (3, 5) | 5 (4, 5) | 5 (4, 5) | 4 | | 4 | | NS | | 5 | |
| 6 | 4 (3, 5) | 1 (1, 1) | 2 (2, 2) | 5 (3, 5) | 5 (4, 5) | 5 (4, 5) | 4 | | 4 | | NS | | 3 | |
| 8 | 4 (3, 5) | 3 (3, 3) | 2 (2, 2) | 5 (3, 5) | 5 (4, 5) | 5 (4, 5) | 4 | | 1 | | NS | | 11 | |
| 9 | 4 (3, 5) | 1 (1, 1) | 2 (2, 2) | 5 (3, 5) | 5 (4, 5) | 5 (4, 5) | 2 | | 4 | | NS | | 12 | |
| 10 | 4 (3, 5) | 1 (1, 1) | 2 (2, 2) | 5 (3, 5) | 5 (4, 5) | 5 (4, 5) | 3 | | 3 | | NS | | 8 | |
| 10 | 4 (3, 5) | 1 (1, 1) | 2 (2, 2) | 5 (3, 5) | 5 (4, 5) | 5 (4, 5) | 3 | | 3 | | NS | | 15 | |
| 12 | 4 (3, 5) | 1 (1, 1) | 2 (2, 2) | 4 (3, 4) | 5 (4, 5) | 4 (4, 4) | 1 | | 4 | | NS | | 6 | |
